# Associations between overweight, obesity, and mental health: a retrospective study among European adults aged 50+

**DOI:** 10.3389/fpubh.2023.1206283

**Published:** 2023-07-18

**Authors:** Gregor Alexander Rindler, Anna Gries, Wolfgang Freidl

**Affiliations:** ^1^Institute of Social Medicine and Epidemiology, Medical University, Graz, Austria; ^2^Division of Physiology and Pathophysiology, Otto Loewi Research Center for Vascular Biology, Immunology and Inflammation, Medical University, Graz, Austria

**Keywords:** mental health, obesity, overweight, long-term, association, body mass index, depression, quality of life

## Abstract

**Background:**

The comorbidities associated with overweight and obesity have been well researched and scientifically proven while their relationship to mental health is still not verified.

**Methods:**

This study is aimed at investigating reciprocal associations between obesity and mental health, and is intended to further analyze possible long-term effects using data from the Survey of Health, Ageing and Retirement in Europe (SHARE). In order to do that, waves 4 and 8, conducted in 2010 and 2019/20 of this survey, were analyzed in a cross-lagged panel approach including 16,184 adult Europeans (50+) using multiple linear regression analysis focusing on the Body Mass Index (BMI), depression status and quality of life (QoL).

**Results:**

Findings yield significant cross-lagged effects in one direction regarding BMI predicting QoL and depression state, whereas depression state and QoL do not significantly predict BMI. Findings include people living with obesity, overweight, and underweight showing significantly decreased levels of QoL as well as increased depression scores compared to people of normal weight over a lag time of 10 years, where people living with obesity indicate the strongest effect.

**Conclusions:**

However, results do not confirm reciprocal associations in the long term. Hence, there is a strong need to carry out further research on this issue.

## 1. Introduction

Obesity is the fifth leading cause of death worldwide (1) and is therefore considered a global epidemic, demonstrating one of the major health-related issues not only in industrialized countries but also in developing countries (2, 3). According to the World Health Organization (WHO), overweight and obesity are defined as a BMI greater than or equal to 25, respectively, a BMI greater than or equal to 30 (4). Over recent decades, however, sedentary lifestyles have increased and calorie-rich foods have been consumed more frequently, leading to a substantial increase in body weight and BMI levels. Besides increased mortality rates and impaired QoL, obesity and its associated comorbidities such as cardiovascular events or diabetes mellitus have a significant impact on health care expenditures.

Additionally, mental disorders such as impairments of mood, thoughts as well as behavioral dysregulation contribute to burden of disease (5). According to the Global Burden of Disease (GBD) study, mental disorders as well as disorders linked to substance use are considered the fifth leading cause of burden with respect to disability adjusted life years (DALYs) (6, 7). Both obesity and mental illness show increasing prevalence rates and are associated with numerous medical complications in vulnerable populations, as shown in several studies (8–11). Presumably, the coexistence of these conditions is more than just a random overlap. However, there is still a lack of knowledge in terms of linking mechanisms (12). People who live with severe mental disorders have a significantly shorter life expectancy of about 20 years compared to the general population. This may be due to a sometimes unhealthy lifestyle and the frequent occurrence of physical diseases (13), including cardiovascular issues (14–16). According to a global systematic review and meta-analysis on lifestyle patterns of people with mental disorders such as schizophrenia, bipolar disorder, and major depressive disorder, sedentary behavior and less physical activity was increasingly observed among those people. About 50% of individuals living with a mental disorder do not follow the general recommendation of at least 150 min of exercise per week (17). Furthermore, people with severe mental illness often show unhealthy eating habits, including a low consumption of fruits and high fiber food, but a high intake of junk food (18, 19). Additionally, people with mental disorders are more frequently heavy smokers than the general population (20–22).

First reports describing increased risks of obesity in patients with mental disease were established by Nicholson in 1946. Accordingly, Nicholson documented emotional tensions and psychoneurosis to be related to obesity (23). Since then, numerous studies on the relationship between these clinical conditions have been carried out leading to predominantly focus on a bi-directional association (24). In addition, individuals who have mental illnesses show a 2- to 3-fold increased risk of obesity. In contrast, people living with obesity are at a 30 to 70% higher risk of developing a mental illness (25). A North-American study found that about 80% of 10,000 people diagnosed with schizophrenia, bipolar disorder or depression were either affected by being overweight or lived with obesity (26). The reason for this high number of patients living with obesity and severe mental illnesses has been widely debated. Several studies state that complex interactions between genetic, environmental, disease-inherent factors, and the side effects of antipsychotic drugs appear to be responsible for gaining body weight (27). Others see the main reason for this increase in antipsychotic adverse events (28–31). It is generally assumed that weight gain, as a side effect of antipsychotic drugs, may emerge from multiple mechanisms, including the neurotransmission of hormones, such as serotonin, histamine and/or epinephrine (32). Active agents of antipsychotics act on neural pathways by modulating their activity. As a consequence, weight gain may be due to the blockade of specific receptors, which has an impact on regulatory mechanisms of appetite and body weight (33). Both antidepressants (34–39) and antipsychotic medications can cause increased BMI levels and may further lead to a greater cardiovascular risk, indicating an exponential rise in BMI scores and metabolic risk (40). Furthermore, different, sometimes unhealthy lifestyles such as smoking, lack of physical activity, and their effects, further complicate causal research (41, 42). Both mental illness and obesity are chronic conditions which are very prevalent. Besides, some examinations point out that these two events are linked to each other (43). Possible associations between obesity and physical and mental ill health have been widely discussed (44). Some examinations point out that individuals with psychiatric disorders are at a higher risk of developing obesity (45, 46) and people with mental illnesses are much more likely to live with either overweight or obesity than the average population (47). Depression, impaired mental health and poor quality of life are often associated with obesity (48). In addition, this co-occurrence seems to be particularly likely when people are affected by physical impairments as well. Thus, the relationship between obesity and mental health seems to be stronger in later life than in early periods of life or middle-aged individuals (43, 49). A systematic review and meta-analysis conducted by Mannan et al. suggests that depressive adolescents are at a 70% increased risk of obesity, and adolescent individuals with obesity are at a 40% greater risk of being depressed (50). The comorbidities related to overweight and obesity have been scientifically confirmed (51, 52), while knowledge of their association with mental health is still deficient (53) and inconclusive (45). Given that most of the studies have investigated the association between mental health and obesity only using measurements made at one time point (43), this work aims to contribute to a better insight into the nature of possible long-term relations, stimulate further discussion, and shed more light on possible reciprocal cause-effect relationships focusing on the mental health constructs depression and quality of life (QoL). Thus, the following hypotheses were formulated:

*Null hypothesis (H_0_): European female and male adults aged 50 years and older who live with either overweight or obesity show no significant positive bi-directional association between their depression score and body mass index (BMI), and also no significant negative bi-directional association between their quality of life (QoL) levels and body mass index (BMI) over a 10 year follow-up period.*

*Alternative hypothesis (H_1_): European female and male adults aged 50 years and older who live with either overweight or obesity show a significant positive bi-directional association between their depression score and body mass index (BMI), and also a negative significant bi-directional association between their quality of life (QoL) levels and body mass index (BMI) over a 10 year follow-up period.*

## 2. Materials and methods

### 2.1. Study design, survey method and samples

In this study, data from the Survey of Health, Ageing, and Retirement in Europe was used to investigate our research question aiming at analyzing a possible bi-directional association between the BMI of male and female individuals from wave 4 and mental health, including QoL and depression status, in wave 8 and vice versa, in the context of a longitudinal analysis using a two-wave cross-lagged panel approach over a defined time lag of 10 years.

The Survey of Health, Ageing and Retirement in Europe was conducted among 50+ individuals and started in 2004, including 11 countries within Europe. Baseline study in wave 1 involved Scandinavia (Denmark, Sweden), central Europe (Austria, France, Germany, Switzerland, Belgium, Netherlands) and the Mediterranean including Greece, Italy and Spain. At the end of 2004, Israel was additionally integrated into the survey. Estonia, Portugal, Slovenia and Hungary joined the third regular panel in wave 4 carried out in 2010, including 58,000 participants constituting the gross data basis for this study. Fieldwork for the eighth wave in October 2019 was interrupted due to the COVID-19 pandemic in March 2020, resulting in a considerably shortened questionnaire. Therefore, the following countries were included: Austria, Belgium, Bulgaria, Cyprus, Croatia, Czech Republic, Denmark, Estonia, France, Germany, Greece, Israel, Italy, Luxembourg, Poland, Spain, Sweden, Lithuania, Latvia, Malta, Finland, Switzerland, Slovenia, Hungary, Netherlands and Slovakia (54) resulting in a gross sample size of 46,733 of which 19,915 participants completed main interviews in the fourth wave.

The target population of the SHARE survey consisted of people who were 50 years of age or older at the time of the surveys and who had their regular residence in the relevant country of the survey. Persons who were either hospitalized, incarcerated, or out of the country for the entire duration of the interview were excluded from the interviews. Other exclusion criteria included insufficient language skills with regard to the respective national language or moving to an unknown address. All persons of a household born in 1960 or earlier (wave 4) and 1969 or earlier (wave 8) were admitted to interviews (55). The participation in the interviews was voluntary and confidential. All collected data from the survey interviews was linked to a wave-specific identifying variable (ID) (54).

All data was collected using computer-assisted personalized interviews (CAPI) and face-to-face interviews. This process was further enhanced by self-completed paper & pencil questionnaires (54). Each interviewer was using a laptop when conducting face-to-face interviews on which the CAPI tool was installed. Due to the need of executing various physical tests, this personalized approach was necessary for the survey. SHARE uses an ex-ante harmonization concept, that is, one general questionnaire is translated into the respective national languages. This is made possible through the use of an internet-based translation tool, which carries out an automatic translation into the CAPI instrument (55). Based on a significant dropout of several respondents over the time period of 10 years who either died, moved from their regular residence, could no longer be contacted, or refused to participate, we finally obtained a net sample of 16,184 from 19,915 individuals who participated in both wave 4 and wave 8 providing complete data without defined missing values for further analysis. Another reason for the withdrawal of participants in the eighth wave from the survey might have been the beginning of the COVID-19 pandemic in 2019/2020, which is why the study was discontinued.

### 2.2. Institutional review board statement

The study was conducted according to the guidelines of the Declaration of Helsinki and obtained approval from the Ethics Committee of the Medical University of Graz, Austria (Document Number 32-305 ex 19/20).

### 2.3. Measures

#### 2.3.1. Central variables

Overweight and obesity were derived from the variable body weight and height of each respondent in the survey and desired BMI values were then calculated according to Quetelet’s formula: Weight divided by height in meters squared, expressed in kg/m^2^ (56). Accordingly, individual categories of BMI were defined (4) representing overweight as a BMI greater or equal to 25, and obesity classified as a BMI greater or equal to 30. Depression status was measured using the EURO-D scale, an instrument for assessing late-life depression in different countries across Europe consisting of 12 items. Respondents can reach a score between 0 and 12, where a higher score indicates a higher degree of depression. Accordingly, scores equal to or higher than 4 represent a case of depression while values less than 4 correspond to the “no depression” category (57). The mental health variable QoL was collected using the CASP-12, a 4-point Likert scale questionnaire, comprising 4 sub-scales, including control, autonomy, self-realization, and pleasure. All 12 items constitute an overall score ranging from a minimum of 12 to a maximum of up to 48, whereby higher scores represent higher levels of QoL (58).

#### 2.3.2. Control variables

It is well recognized that both social and economic factors have an impact on people’s mental health status, whereby natural and man-made problems and situations may affect their resilience (59, 60). Furthermore, social aspects may have an impact on gaining body weight (61). The influence of control variables such as age, sex, living situation, and educational level, acting as confounding factors, was investigated using multiple linear regression analysis.

### 2.4. Psychometric properties

Internal consistency reliability of mental health measures was calculated using Cronbach’s alpha. The EURO-D scale which was applied in wave 4 and wave 8 resulted in values ranging from 0.62 to 0.78 and proved to be rather moderately internally consistent in all included European countries (62). Moreover, the abridged CASP-12 scale indicates sufficient Cronbach’s alpha scores between 0.56 and 0.76 across different countries (63).

### 2.5. Preparation and data cleaning

The first step was to match relevant SHARE data sets including central characteristics from waves 4 and 8 as well as sociodemographic variables. The matching procedure was accomplished using a person identifier variable as a key variable. Given that some cases yielded data on wave 4 but not on wave 8, all following calculations, including respective hypotheses tests, refer entirely to the cases in which all data was available. Remaining, non-paired cases were excluded using a filter variable. The age of respondents was transformed into categories representing three age groups: “50–59”, “60–74”, “75+”. Collected information on the “living situation” of respondents was transformed into a binary coded variable for “living alone,” and “living with a partner.” Furthermore, we created dummy variables of the categorical predictors, including sex, BMI, and educational level to allow the analysis of their impact on the respective outcome variables. Responses such as, “refuse,” “do not know,” “implausible or suspected wrong,” were defined as missing values and were excluded from the analysis.

### 2.6. Statistical analysis

**Figure 1:**
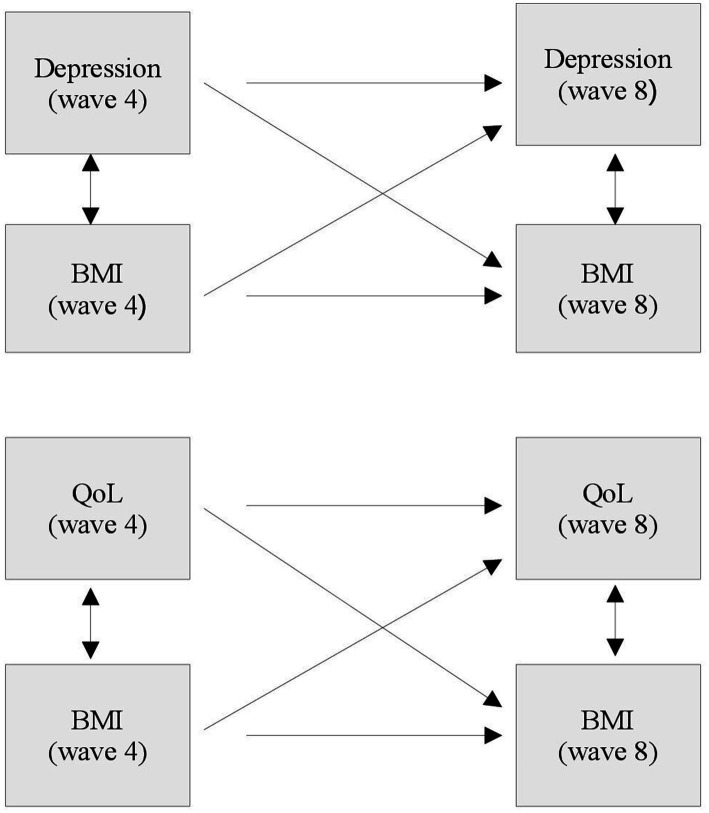
Overview of synchronicity, stability associations and cross-lagged pathways between central constructs: depression (EURO-D), QoL (CASP), and body mass Index (BMI). Single-headed arrows represent synchronous (cross-sectional) paths; double-headed arrows demonstrate stability (horizontal) and cross-lagged (diagonal) associations.

Statistical analysis was entirely performed using IBM SPSS statistical software (Version 26). Addressing the long-term effects, according to our hypothesis, was accomplished using multiple linear regression analysis to define associations between BMI categories and mental health variables, depression and QoL. The level of significance (value of *p*) was defined at *p* < 0.05 (*α* = 5%). In order to evaluate the association of predicting variables from wave 4 on outcome variables in wave 8, a two-wave-cross-lagged panel approach (64–66) with a time lag of about 10 years was conducted in the context of a longitudinal analysis. Central variables, BMI, depression status and QoL were measured at two different time points (wave 4 and 8) leading to six possible relations (65), which were analyzed using multiple regression analysis to determine stability (autoregressive) and cross-lagged effects, whereby synchronicity of constructs at both time points was assumed. Synchronicity, stability and cross-lagged associations of central variables in waves 4 and 8 result in two synchronous (cross-sectional), two stability (autoregressive), and two cross-lagged relations (65). In order to investigate the stability of the relation between BMI values at two time points, BMI was used as a metric predictor. Accordingly, autoregressive and cross-lagged effects between different measures in waves 4 and 8 were calculated simultaneously to determine the size of stability of each construct and reciprocal associations over time (Figure 1). Accordingly, the analysis of stability and cross-lagged effects resulted in 3 models (Table 1).

**Table 1:** Overview of models analyzing stability and cross-lagged associations between central constructs.

Model	Stability path^a^	Cross-lagged path^b^
1	CASP CASP	underweight^b^ CASP
DV: CASP		overweight^b^ CASP
		living with obesity^b^ CASP
2	EURO-D EURO-D	underweight^b^ EURO-D
DV: EURO-D		overweight^b^ EURO-D
		Living with obesity^b^ EURO-D
3	BMI BMI	CASP BMI
DV: BMI		EURO-D BMI

a Autoregressive associations of constructs between wave 4 and wave 8.

b Crossed associations over a 10 year time lag.

BMI, body mass index; CASP, QoL, EURO-D, depression; DV, dependent variable.

Based on these results, the impact of sociodemographic variables, including age, sex, living situation and educational level (ISCED-97) on respective outcome variables was analyzed. Additionally, further multiple linear regression analysis adjusting for sociodemographic characteristics was conducted.

## 3. Results

### 3.1. Demographics and health characteristics of participants

In the longitudinal analysis, 16,184 people (aged 50 and above) were included, consisting of 9,428 (58.3%) female and 6,756 (41.7%) male respondents. The mean age of the participants was 63.6 years (SD = 8.1). The youngest participants were 50 and the oldest was 92 years of age. Most of the participants were from Estonia (13.1%) and the Czech Republic (10.1%). The majority of respondents reported living together with their partner (74.9%). In addition, 37.6% of the sample population indicate an upper secondary education (level 3) (Table 2).

**Table 2:** Distribution of demographic characteristics in SHARE (*n* = 16,184).

Country	*n*	%
Austria	1,216	7.5
Germany	552	3.4
Sweden	739	4.6
Netherlands	906	5.6
Spain	701	4.3
Italy	945	5.8
France	1,588	9.8
Denmark	928	5.7
Switzerland	1,581	9.8
Belgium	1,153	7.1
Czech Republic	1,632	10.1
Poland	480	3.0
Hungary	679	4.2
Slovenia	967	6.0
Estonia	2,117	13.1
Sex		
Male	6,756	41.7
Female	9,428	58.3
Age group		
50–59	5,631	34.8
60–74	8,801	54.4
75+	1752	10.8
Partner in household		
Yes	12,116	74.9
No	4,068	25.1
Level of Education*		
ISCED-97 Code 1	2,262	14.0
ISCED-97 Code 2	2,906	18.0
ISCED-97 Code 3	6,090	37.6
ISCED-97 Code 4	960	5.9
ISCED-97 Code 5	3,837	22.9
ISCED-97 Code 6	129	0.8

*Code 1–6 of Education refers to the Level of 1–6 of Education: Primary education/first stage of basic education (ISCED-97 Code1), lower secondary education/s stage of basic education (ISCED-97 Code 2); upper secondary education (ISCED-97 Code 3), post-secondary, non-tertiary education (ISCED-97 Code 4), first stage of tertiary education (ISCED-97 Code 5), second stage of tertiary education (ISCED-97 Code 6).

The mean BMI score of the study population was 27.0 kg/m^2^ (SD = 4.7). The majority of respondents were overweight (42.1%) followed by normal weight (35.2%) and people living with obesity (21.8%) – underweight people only account for a minority of 0.9%. The mean depression score according to the EURO-D scale was 2.2 (SD = 2.1) reflecting an overall non-depressed state of the population. After measuring QoL using the CASP-12 scale ranging from 0 to 12, mean values and standard deviations were calculated, indicating a mean QoL score of 38.4 (SD = 5.9) indicating a high level of QoL within the study population (Tables 3, 4).

**Table 3:** Distribution of BMI categories in SHARE (wave 4).

BMI Category	*n*	%
underweight	145	0,9
normal weight	5,701	35.2
overweight	6,815	42.1
living with obesity	3,523	21.8

**Table 4:** Descriptive statistics of variables in SHARE (*n* = 16,184).

Variables	Mean	SD
BMI	27.0	4.7
CASP Index	38.4	5.9
Depression (EURO-D)	2.2	2.1
Age	63.6	8.1

### 3.2. Results of multiple linear regression analysis

All three models demonstrate good to high fit to the data. The first model (model 1) was significant in terms of the stability of QoL (CASP) scores and BMI categories predicting QoL over time in the eighth wave (*F* (4,16,179) = 1575.441, *p* < 0.001, *R*^2^ = 28.0%). As a result, QoL scores in wave 4 seem to significantly predict QoL scores (autoregressive path) at a second time point in wave 8 (*β* = 0.515, *p* < 0.001). This mental health characteristic indicates high stability over a lag time of about 10 years. Cross-lagged analysis of BMI categories predicting QoL indicates that people living either with overweight, obesity or underweight show significantly decreased levels of QoL in comparison to respondents of normal weight, where people living with obesity show the strongest effect (*β* = −0.084, *p* < 0.001).

Our second model (model 2) yields a significant result as well (*F* (4,16,179) = 820.656, *p* < 0.001, *R*^2^ = 16.8%). The Depression score proved significantly stable in the long term (*β* = 0.403, *p* < 0.001). The analysis of cross-lagged associations yields that BMI categories, underweight, overweight and obesity significantly predict depression status over time, resulting in increased scores of depression compared to people of normal weight. Again, with the strongest effect among individuals who live with obesity (*β* = 0.063, *p* < 0.001).

The third model (model 3) was also significant (*F* (3,16,180) = 820.656, *p* < 0.001, *R*^2^ = 61.2%) demonstrating a significantly stable association regarding the autoregressive path. The BMI score in wave 4 predicts the BMI score in wave 8, indicating high stability (*β* = 0.782, *p* < 0.001), whereas depression state and QoL do not significantly predict BMI over time (Table 5).

**Table 5:** Overview of stability and cross-lagged coefficients-unadjusted linear regression model (*n* = 16,184).

Variables	CASP (wave 8)		EURO-D (wave 8)		BMI (wave 8)	
	*β*	*p*	*β*	*p*	*β*	*p*
CASP Index	0.515^b^	<0.001	–	–	0.000^c^	0.950
EURO-D	–	–	0.403^b^	<0.001	0.006^c^	0.266
underweight^a^	−0.026^c^	<0.001	0.020^c^	0.005	–	–
overweight^a^	−0.044^c^	<0.001	0.026^c^	0.001	–	–
obesity^a^	−0.084^c^	<0.001	0.063^c^	<0.001	–	–
BMI^d^	–	–	–	–	0.782^b^	<0.001
Adjusted *R*^2^	28.0%		16.8%		61.2%	

a Normal weight is a reference category.

b Stability (autoregressive) coefficients over 10 year time lag.

c Cross-lagged coefficients over 10 year time lag.

d Metric scaled.

–, not applicable.

### 3.3. Results of multiple linear regression analysis adjusted for sociodemographic confounders

In order to investigate the impact of sociodemographic covariates on respective outcome variables, we performed a second run of analysis adjusting for baseline characteristics, including sex, age, living situation, and educational level. In addition, cross-lagged associations between central constructs and stability correlations were retested according to models 1 to 3 described above, including confounding predictors.

The first model examining the effect of BMI categories and confounding factors on QoL was significant (*F* (12,16,171) = 647.113, *p* < 0.001, *R*^2^ = 32.4%). The analysis reveals that respondents of older age indicate a significantly lower QoL, which comparably demonstrates the strongest effect among sociodemographic characteristics within this model in terms of predicting QoL (*β* = −0.166, *p* < 0.001). Moreover, our first adjusted model provides no evidence that sex of respondents significantly affects QoL over time. The education of respondents seems to significantly affect QoL. In fact, the higher the level of education the higher the QoL in the long term, while educational level 5, referring to tertiary education, represents the strongest educational predictor of QoL (*β* = 0.118, *p* < 0.001). Individuals who live with their partners show significantly higher QoL than those who live alone (*β* = 0.032, p < 0.001).

The second model examined the impact of confounding factors on depression scores, yielding a significant result (*F* (12,16,171) = 345.542, *p* < 0.001, *R*^2^ = 20.3%). Similar to model 1, age of respondents seems to play a significant role in predicting depression scores over time. People of older age show higher depression scores over a time-lag of about 10 years (*β* = 0.138, <0.001). Furthermore, females significantly indicate increased depression scores compared to males (*β* = 0.091, <0.001). The level of education seems to affect depression scores. More precisely, higher educated individuals indicate significantly lower depression levels over a 10 year period in contrast to individuals with low education. Again, tertiary educated individuals, who belong to educational level 5, show the strongest effect in terms of decreased depression scores (*β* = −0.094, *p* < 0.001), while living with a partner has no significant effect on depression status compared to living without a partner.

The third model proved to be significant as well (*F* (11,16,172) = 2421.248, *p* < 0.001, *R*^2^ = 62.2%). Interestingly, older respondents seem to have significantly lower BMI scores (*β* = −0.100, *p* < 0.001), whereas sex has no significant effect on BMI scores. With regards to education, in contrast to QoL and depression status, education levels 5 and 6 (tertiary education) make a significant contribution to the prediction of BMI (*β* = −0.028, *p* < 0.001; *β* = −0.013, *p* = 0.008). Thus, tertiary education seems to be negatively associated with BMI, indicating that tertiary educated individuals show decreased BMI scores. Overall, living situations, that is “living with a partner,” does not significantly predict BMI (Table 6).

**Table 6:** Overview of stability and cross-lagged coefficients-adjusted linear regression model (*n* = 16,184).

Variables	CASP Index (wave 8)		EURO-D (wave 8)		BMI (wave 8)	
	*β*	*p*	*β*	*p*	*β*	*p*
CASP Index	0.487^c^	<0.001	–	–	−0.001^f^	0.856
EURO-D	–	–	0.378^e^	<0.001	0.003^f^	0.567
BMI category						
underweight^d^	−0.025^f^	<0.001	0.017^f^	0.014	–	–
overweight^d^	−0.031^f^	<0.001	0.025^f^	0.002	–	–
obesity^d^	−0.077^f^	<0.001	0.060^f^	<0.001	–	–
BMI^g^	–	–	–	–	0.779^e^	<0.001
Sex^a^	−0.009	0.168	0.091	<0.001	−0.004	0.414
Age	−0.166	<0.001	0.138	<0.001	−0.100	<0.001
Education						
ISCED-97 code 2^b^	0.025	0.006	−0.047	0.001	−0.002	0.720
ISCED-97 code 3^b^	0.089	<0.001	−0.077	<0.001	−0.004	0.588
ISCED-97 code 4^b^	0.052	<0.001	−0.050	<0.001	−0.000	0.997
ISCED-97 code 5^b^	0.118	<0.001	−0.094	<0.001	−0.028	<0.001
ISCED-97 code 6^b^	0.014	0.037	−0.010	0.153	−0.013	0.008
Living situation						
Partner in household	0.032	<0.001	0.006	0.434	−0.010	0.043
Adjusted *R*^2^	32.4%		20.3%		62.2%	

a Female gender is a reference category.

b Primary educational level is a reference category.

c Living with a partner is a reference category.

d Normal weight is a reference category.

e Stability (autoregressive) coefficients over 10 year time lag.

f Cross-lagged coefficients over 10 year time lag.

g Metric scaled.

–, not applicable.

In summary, the stability of central constructs proved significant over a 10 year time period in all three models. Significant cross-lagged effects can be observed in one direction between BMI predicting QoL and depression state, but not vice versa. Hence, people living with underweight, overweight or obesity show lower QoL and higher depression scores than people of normal weight, whereas obesity indicates the strongest effect regarding the prediction of both QoL and depression state over a lag-time of 10 years. Accordingly, results do not confirm any reciprocal association. The depression state and QoL do not significantly predict BMI in older adults. Moreover, attention has to be paid to the rather small strength of the respective cross-lagged effects.

Sociodemographic predictors such as educational level and age of respondents seem to significantly affect QoL, depression state, and BMI over time. Higher education seems to be associated with lower BMI and depression scores. More highly educated people show greater QoL levels than those with lower levels of education. Female gender obviously predicts increased depression scores, whereas no significant impact on QoL and BMI can be observed. The older age of respondents also proved to be a significant predictor for increased depression scores and decreased QoL, but is negatively associated with BMI scores, implying that older individuals display lower BMI scores. However, the effects are rather small.

## 4. Discussion

This study is grounded on the assumption that European female and male adults aged 50 years and older who live either with overweight or obesity show a significant positive bi-directional association between their depression score and body mass index (BMI), and a significant negative bi-directional association between their quality of life (QoL) levels and body mass index (BMI) over a 10 year follow-up period. Accordingly, this investigation was performed using longitudinal data from older individuals aged 50 years of age or above to determine possible associations in both directions using a two-wave cross-lagged panel approach. In order to investigate the associations between mental health constructs and overweight or obesity over a time-lag of about 10 years, multiple regression analysis was conducted focusing on answering the hypothesized research question. Mental health characteristics were measured by means of well validated questionnaires that demonstrate good to excellent psychometric properties and BMI scores were calculated constituting the foundation for our analysis.

With regard to our main research hypothesis, the findings of the investigation of possible associations between BMI levels and mental health variables yields indications of significant small effects in terms of predicting QoL and depression scores implying higher depression and lower QoL scores among those people who live with underweight, overweight or obesity compared to persons of normal weight. Accordingly, people living with obesity demonstrate the strongest effect regarding the prediction of both QoL and depression status. Despite significant results, the strength of these effects is rather small and should therefore be interpreted with caution in terms of confirming any considerable association in the long term. The hypothesis of a one-directional association (25, 43, 48, 50) was grounded on the assumption that female and male adults aged 50+ who live with either overweight or obesity indicate a significant positive association between their BMI predicting depression score and a negative association between participants´ BMI and quality of life (QoL) levels in a 10 year follow-up. As a result, a significant one-directional cross-lagged effect can be observed regarding BMI scores predicting QoL and depression status. People who live with overweight and obesity are more likely to show increased depression scores and decreased QoL. Furthermore, regression analysis reveals that neither depression nor QoL predict BMI. However, we were not able to confirm any significant reciprocal associations between mental health and overweight or obesity.

Obviously, certain sociodemographic characteristics such as age, level of education, and sex are associated with mental health characteristics and BMI. In addition, living with a partner seems to slightly increase QoL and decline BMI. Female gender, in particular, seems to significantly predict increased depression scores. Another result of our study indicates that respondents of older age are more likely to show decreased BMI scores, which is consistent with suggestions by Wysokiński et al. who postulate that many older, even healthy individuals, experience weight loss, which may be associated with an age-related reduced food intake resulting from dysregulated appetite, especially among people of very advanced age referred to as “anorexia of aging” (67). The negative association between older age and reduced BMI scores, according to our study, may be due to the fact that our sample population also included some respondents of very advanced age. However, to the best of our knowledge, there is no clear evidence available for younger populations. As suggested by previous research (68, 69), anorexia of aging and the related loss of weight typically occurs among the older adults above 65 years of age who are either hospitalized, in long-term care facilities, or live with neurological disorders or inflammatory diseases. This effect is therefore thought to be an age-related condition emerging from metabolic changes, loss of appetite, and frailty, among other things (70). As a consequence, the decrease of weight and body fat is considered a typical late-life phenomenon (71). According to several studies on the risk of developing depression, demographic conditions such as female gender (72, 73) and older age (74, 75) are recognized risk factors for the development of this mental health condition. Regarding sociodemographic parameters, the findings of this study turned out to be in line with the results of previous studies (59, 76). In particular, social determinants including age, sex, and educational attainment seem to be associated with people’s mental health (60). In line with our expectations, and consistent with prior research (72, 73), female gender appears to be more strongly associated with reporting higher depression scores compared to males, whereas sex of participants has no significant impact on QoL.

The main finding of this study that BMI significantly predicts depression state and quality of life levels, is partially consistent with suggestions from a systematic review and meta-analysis conducted by Mannan et al. who investigated bi-directional associations between obesity and depression (50). The results obtained by Mannan et al. suggest a significant depression-obesity link regardless of the direction of association, although the strength of association turned out to be more significant in terms of depression leading to obesity than vice versa (50). Nevertheless, findings obtained from multiple regression analysis are not in line with the suggestions made by McElroy et al. and Pickering et al. who stated that mentally ill individuals are at a higher risk of developing overweight and obesity (45, 46) since no significant link between mental illness leading to overweight or obesity can be observed with respect to depression status and QoL level. Yet, our findings indicate some similarities with the results from Luppino et al. who postulated that low QoL and depression often co-occurs with obesity and may be highly prevalent among older individuals who experience impaired physical conditions (48).

Furthermore, a meta-analysis of several longitudinal studies conducted by Luppino et al. found that obesity and depression are reciprocally associated (48). As a result, our findings are consistent with the suggestions by Luppino et al. who claim that depression, mental health issues, and poor quality of life (QoL) are frequently related to obesity (48). Moreover, our findings are in line with the suggestion by (43, 49) indicating that the association between obesity and mental health is more pronounced in older individuals (43, 49). In this regard, our results suggest that people at older age living with obesity and overweight indicate decreased QoL and higher depression levels in comparison to people of normal weight. Furthermore, results from multiple regression analysis are consistent with the statements of Avila et al. claiming that obesity is significantly associated with reduced QoL. In accordance with that, mental illnesses such as depression seem to be significantly associated with obesity and may also lead to a considerable decrease in QoL. In the case of co-occurrence of these two conditions, effects seem to amplify significantly. Despite the existing public awareness and corresponding efforts made, the rising prevalence of both mental illness and obesity is still a fundamental issue (12).

Correll et al. suggest that the majority of mentally ill individuals live with obesity, respectively overweight, hinting at a possible link between mental illness and obesity (26). Accordingly, our findings agree with previous research from Corell et al. in terms of identifying a significant relationship between BMI and depression status (26) in the sense that obesity leads to increased depression scores. However, results obtained from our investigation also reveal that people living with underweight show significantly reduced QoL and increased depression scores as well, even though the effect size is at its lowest here.

The core finding of this investigation is in line with the suggestions by De Hert and colleagues who postulate that people living with obesity are more likely to have a mental illness (25). However, this concordance, according to our result, may only apply to the constructs depression and QoL. Considering that the relationship between obesity and mental health seems to be more pronounced in older adults than in younger populations, our major finding, that is, BMI is significantly associated with increased mental health issues, is consistent with the claims made by (43, 49). However, when interpreting the results, especially with regard to the one-directional association between BMI and mental health variables, it should be taken into account that the participants were already over 50 years of age, and we do not have reliable data about the onset of obesity. In addition, the dichotomization into depressed/non-depressed does not account for the severity of depression. Another important point is the effect of taking psychotropic medication, that is, antidepressants (34–39) and antipsychotics, which are considered major leading factors causing obesity and may contribute to the outcome of this study. Hence, this might be a possible explanation for our result indicating BMI predicting mental health but not vice versa. Additionally, another explanation could be the fact that many respondents of advanced age with a lower BMI were included in our study, which could also have an impact on this outcome.

### 4.1. Limitations and strengths of this study

In contrast to the performance of randomized control trials (RCTs) for investigating causal effects among study populations, longitudinal studies of observational data such as the Survey of Health, Ageing and Retirement in Europe (SHARE) offers the opportunity to study long-term, and hypothesized bi-directional associations over a defined time-lag. Although results can never prove complete causality. Nevertheless, this study provides a longitudinal design that is highly desirable in order to investigate cause-effect associations between mental health and overweight over time. Despite a large sample size, including respondents from all over Europe, this study does not account for further possible confounding factors such as physical activity, eating behavior, physical comorbidities or the use of psychotropic medication, which may substantially contribute to causing obesity and metabolic side effects (40).

In fact, information on the intake of psychotropic drugs of respondents may have an impact on results considering that prior research (34–39) suggests that certain antidepressants are suspected to potentially increase the risk of weight gain, in addition to antipsychotic drugs. Following the suggestions from an early review conducted by Fava, older antidepressants such as mirtazapine, within the subgroup of tricyclic antidepressants (TCAs), and monoamine oxidase inhibitors (MAOIs), may more frequently increase body weight compared to the newer generation of selective serotonin reuptake inhibitors (SSRIs) (35), which is also in accordance with suggestions by Serretti and Mandelli who claim in their meta-analysis that mirtazapine, among other antidepressants, is considered a cause of weight gain (36, 37, 39). Gaining weight from these medications may in some cases result from complex hormonal mechanisms, including the antihistaminergic effect of both antipsychotics and certain antidepressants on H_1_ histamine receptors, and imbalances of the orexigenic and anorexigenic hormones ghrelin and leptin (77–79). Yet, it should be considered that the mechanisms through which antidepressants may potentially be associated with weight gain are unclear and poorly understood (80) assuming that these findings may not warrant causal inference, while the effect of residual covariates possibly contribute to overrate this association (81).

According to Rogosa, investigating long-term effects using a two-wave cross-lagged procedure is not useful for determining causal inference (82), whereas Selig and Little suggest that this panel design may generate more insight, better understanding of longitudinal associations, and stimulate further research (83). Another limitation of this study is that the anthropometric characteristic body weight was self-reported, which carries the risk of response bias. Moreover, we were not able to account for weight bias and stigmatization of people living with obesity, which obviously may affect people’s mental health status. Individuals who live with obesity often experience weight bias and being targeted for jokes because of their body weight, especially via the media, which further promotes the stereotype that larger body size does not comply with the norm, and is socially not acceptable (84).

In fact, one strength of this study is that the analysis is based on observational data derived from a large-scale panel approach which may significantly contribute to generating an adequate sample population encompassing the older adults in their private household setting. The analysis of longitudinal data provides a reliable tool for identifying cause-effect- and bi-directional associations in the long term. Since data collection was performed on people of the general population from 15 European countries, the sample population is assumed to be representative and provides an appropriate sample size yielding generalizable results.

### 4.2. Outlook, implications for the future

Given that both mental illness and overweight are public health issues of great importance, gaining more insight into the complexity of their relationship and exploring the underlying nature of these two entities seem to be crucial in respect of disease prevention and developing innovative treatment strategies considering that mental health issues have often been overlooked in the past (85). The findings of this study should initiate further research focusing on this subject, accounting for links between mental health issues and their impact on body weight, and vice versa, particularly, in the older adults among whom overweight and obesity is highly prevalent. Thus, integrating mental health status into treatment strategies following a biopsychosocial approach and implementing new evidence in clinical settings could improve future treatment and prevention strategies.

In this regard, this study should encourage researchers to set up innovative investigations addressing associations between mental health and obesity, and further analyze interactions between common mental illnesses such as depression and anxiety, with respect to overweight and obesity, particularly with regard to bi-directional long-term effects and corresponding casualties in terms of a cause-effect principle. In fact, evidence on associations between mental health and obesity is still inconclusive (45) and most of the studies that have been carried out in the past are based on a cross-sectional approach, while prospective studies using repeated measurements of both mental illness and overweight are scarce. In particular, further large-scale investigations are required in order to provide profound studies by which potential cumulative effects can be analyzed and discussed in a broader sense. Moreover, generalizability of previous findings needs to be verified (43). Nowadays, both mental illness and obesity are among the greatest global public health challenges. Exploring and understanding the underlying nature of these complex relationships is crucial and highly desirable, considering that the associations between obesity and mental health highlight the importance of addressing both physical and mental health when treating obesity. By addressing mental health concerns, individuals with obesity may be better equipped to manage their weight and improve their overall health and well-being.

### 4.3. Contribution to the field

This work is intended to create stronger awareness and to stimulate further discussion on this issue. In particular, this research provides multiple measuring points within a panel approach, which is highly necessary to assess reciprocal interactions. Most of the prior research focusing on the relationship between obesity and mental health predominantly determines synchronous effects of a given population leading to results that are generally rather inconsistent (43). Our investigation provides a longitudinal design that allows us to estimate reciprocal interactions, which is quite rare in view of the existing evidence. Since it is well known that both obesity and mental health issues do often occur among older individuals, indicating a highly prevalent issue, our findings provide valuable statements about the associations over a long period of time, and hint to possible causal influences that need to be further analyzed by future research activities. Moreover, most of the studies carried out previously yielded results that do not allow an overall conclusion to be drawn for all age groups, which may have an impact on respective results (48). This study contributes to obtaining a more detailed picture of long-term effects and interactions among older people, which is essential in terms of determining the direction of the relationship. On this foundation, treatment strategies and prevention programs could be further improved by identifying potential risk factors, considering that obesity may be a risk factor for the development of depression, interventions that target overweight and obesity may improve the prevention of depression and low QoL. Furthermore, the results of our investigation should serve as an important foundation for clinical practice suggesting that clinicians and professional care providers should account for mood monitoring in patients living with overweight and obesity, especially when treating older patients with long-term obesity, which may further facilitate the establishment of treatment guidelines.

In addition, we hope that the findings of this study strengthens public awareness about the importance of addressing both obesity and mental health in order to reduce stigmatization and encourage affected individuals to seek treatment. This work should further contribute to identifying early signs of pathological developments and improving interventions for people at a higher risk. We really hope that this research stimulates further scientific discussions and investigation to verify the nature of the relationship between mental health and obesity. Based on our findings, we do believe that further epidemiological research is warranted to determine these underlying mechanisms, notably with regard to possible moderators and confounding factors within the European population. Finally, we hope that our findings inform policy decisions related to public health.

## 5. Conclusion

In conclusion, cross-lagged, one-directional associations between BMI predicting mental health characteristics depression and QoL can be confirmed over a 10 year period. However, this study does not verify any considerable bi-directional associations in the long term corresponding to previous investigations on this topic (12, 45, 46, 48, 50). Even though models show significance regarding explained variances, overall corresponding beta coefficients proved to be rather small. These findings should be taken as weak evidence of a possible association, although this is not proof of a causal relationship. Generally, results should be seen as an impetus for further research on this topic. Gaining knowledge, not only among older populations, but also in terms of detecting early onsets of pathologies among younger populations involving children and adolescents seems to be crucial for preventing illnesses.

## Data availability statement

The original contributions presented in the study are included in the article/Supplementary material, further inquiries can be directed to the corresponding author.

## Ethics statement

The studies involving human participants were reviewed and approved by the Ethics Committee of the Medical University of Graz, Austria (Document Number 32-305 ex 19/20). The patients/participants provided their written informed consent to participate in this study.

## Author contributions

GR, AG, and WF: conceptualization and writing—review and editing. GR and WF: methodology, formal analysis, interpretation of data, and visualization. GR: writing—original draft preparation. WF: supervision and project administration. All authors contributed to the article and approved the submitted version.

## Conflict of interest

The authors declare that the research was conducted in the absence of any commercial or financial relationships that could be construed as a potential conflict of interest.

## Publisher’s note

All claims expressed in this article are solely those of the authors and do not necessarily represent those of their affiliated organizations, or those of the publisher, the editors and the reviewers. Any product that may be evaluated in this article, or claim that may be made by its manufacturer, is not guaranteed or endorsed by the publisher.

## Supplementary material

The Supplementary material for this article can be found online at: https://www.frontiersin.org/articles/10.3389/fpubh.2023.1206283/full#supplementary-material
